# Reviews of Books

**Published:** 1923-02

**Authors:** 


### REVIEWS OF BOOKS.

### MAGIC, ANTHROPOLOGY, AND RELIGION.

**The Golden Bough. A Study in Magic and Religion.** By SIR JAMES GEORGE FRAZER, F.R.S., F.B.A. Abridged Edition. (Macmillan; 18s. net.)

Sir James Frazer's great work—the most famous of all books upon the evolution of religion—fills twelve large volumes and costs several pounds. So great a monument of learning and patience, so vast and important a contribution to our knowledge of the inter-relations of magic, anthropology and religion, deserved to be more readily and more cheaply accessible, and its author has placed every intellectual man and woman under an obligation by abridging it upon the heroic scale—as it stands in this edition something like three-fourths of the original must have been omitted. Yet, the work has been so deftly done that, save for the specialist student, nothing has been lost. It is true that all the notes and references have been omitted and expositions condensed, but ample evidence for the illustration of the leading principles of the book has been retained. Nowhere does new matter supersede old, nor is there any modification of the opinions expressed in the last edition. The reader thus gets the real "Golden Bough," though he must needs go without many of the myriads of strange and curious facts upon which Sir James Frazer has built up his views, and even so the book, with its index, runs to more than 750 large pages. The picturesque and arresting opening chapter—that which describes the King of the Wood, "the ghastly priest who slew the slayer, and shall himself be slain," dwelling by the side of the little woodland Lake of Nemi in the Alban Hills—is preserved intact, and Turner's idyllic picture of the classic lake still, as before, forms the frontispiece. The author has earned the thanks of every cultured reader of the English language for this remarkable abridgment, while the enterprise of the publishers in producing it so handsomely and so cheaply will, we may not doubt, produce its own reward.

### THE SURGEON'S PITFALLS.

**Mistakes and Accidents of Surgery.** By HAROLD BURROWS, C.B.E., M.B., B.S. (London), F.R.C.S., Assistant Surgeon to the Royal Portsmouth Hospital. (Ballière, Tindall & Cox; 10s. 6d. net.)

Besides the surgeon there are so many workers in hospital life whose duties bring them into contact, direct or indirect, with the operating theatre that Dr. Burrows's book has a far wider interest than might, at first sight, be imagined. Emphatically, it is not a book for "the man in the street," nor is it intended to be. In spite of determined efforts by the daily newspapers to extract the sensational from its pages, the fact remains that Mr. Burrows has made a strictly professional appeal to those of his colleagues who care to read and earnestly to think. Whether they like his outspoken comments or not, we have no doubt that he will hold their attention. Two main considerations come before the reader, whatever part he may customarily play in hospital life. The first concerns danger-points generally. The second involves reflection upon sundry accepted practices of the day. Danger-points do not come with unusual cases only. The patient complaining of acute abdominal pain is a common enough visitor in any hospital, yet this pain considered by itself, as an isolated fact, has hardly any diagnostic significance at all :—

"Pain in the abdomen may be due to extra-peritoneal disease. Furthermore, in some cases of peritonitis there may be little or no pain. Probably most of us, at one time or another, have made false steps through attributing too much diagnostic value to stomach-ache and vomiting, while paying too little attention to the remaining features of the case. That is why we sometimes perform laparotomy for the cure of symptoms which are really due to locomotor ataxia, lead colic, empyema and pericarditis. We fail to observe the case as a whole, our minds are being held in thrall by certain individual signs upon which we wrongly think we can rely."

As typical of the second aspect of these reflections, we read :—

"It is curious how much prejudice comes into play when treatment of parturition is under consideration. . . . We see a peculiar hesitation and reluctance in the medical profession to allow women to have the benefit of Cæsarean section. Possibly some confusion of mind is responsible for this backwardness. For there are two kinds of Cæsarean section. The premeditated clean operation is one thing. The unforeseen, haphazard procedure . . . is another. I believe the attendance at our gynæcological clinics would be halved were a more progressive spirit to be adopted towards the advantages of Cæsarean section in all cases in which distocia might otherwise occur."

It is not to be expected that even such mild criticism as this will go unchallenged either within or without the profession. Nevertheless, it is just such comments as these which tend to bring facts to the surface. We are glad also to see that, although the bulk of the book is devoted to pitfalls, avoidance of which depends upon the surgeon's personal endeavours, the errors of his various assistants are not spared. The aseptic "ritual" of the theatre is displayed in its other aspect—the negation of all individual intelligence. The rigid rules of asepsis can never be broken with impunity, but Mr. Burrows urges, rightly we think, a little more pliability in selecting and adapting the means to our particular end. In any case, "the fewer the individuals employed at an operation, the fewer the sources of aseptic fallacy" is a dictum which many hospitals might consider. Emergency lighting of theatres comes in, too, for comment. Cases in which the sac of a strangulated hernia has just been opened when out go all the electric lights in the district may not be of frequent occurrence, but at least such an accident reminds us of the need for dual lighting equipment.

It must be remembered that ignorance is, of course, merely a relative term. There is no exact dividing line between knowledge and the lack of knowledge. Therefore it is good mental exercise to decide which of the author's many instances fall above and which below the line of legitimacy. In some cases it is relatively simple. For example :—

"A married woman was sent to hospital with a note from her doctor in which he stated that she was 'suffering from retention of urine, probably due to an enlarged prostate.' At any rate, the case 'was not one of ordinary stricture,'

the district nurse having been able to draw off the urine with a rubber catheter. The patient had previously, he added, undergone hysterectomy. As a matter of fact, she was suffering from retroversion of the pregnant uterus."

On the other hand, what is one to say of such a rare instance as this :—

"An elderly patient comes with certain abdominal pains, and on examination we find a hard, nodular mass in the right iliac fossa. We judge this to be a carcinoma of the large bowel, and as the mass is fixed and there are evidences of involvement of the encompassing structures, we decide that the prospect of any successful operative interference is unfavourable. . . . Time drifts by and the patient leaves the town. . . . By this time a sinus has formed in connection with the tumour. . . . The disease turns out to be actinomycosis, and our reputation suffers a reverse."

Why does the medical man make so many errors of judgment ? Partly, no doubt, because he lacks experience and knowledge, but often in the surgeon's case because the judgment of a tired man is of little value. Not only this, for there is a dangerous form of bias which accompanies all concentration of thought. With the mind's eye fixed intently upon one point, the remaining field of vision can never be crystal clear. As these mistakes are read of in cold print they all seem readily avoidable, and yet, as we know, are readily committed. The surgical reader may feel that he is being told much that he already knows. Yet he must remember that in over-confidence lies the greatest mistake of all, and we congratulate Mr. Burrows, not only upon charting the simpler of surgery's pitfalls, but also upon rendering a signal service to the surgeons of the future and to their assistants.

### DR. RADCLIFFE'S CANE.

**The Gold-Headed Cane.** By WILLIAM MACHMICHAEL, M.D. New edition, with an introduction and annotations by George C. Peachey. Illustrated. (Henry Kimpton; 18s. net.)

It is somewhat remarkable that a book which professes to do no more than tell, in autobiographical form, the story of so small a matter as a gold-headed cane should have passed through five editions in little more than a hundred years. It might have been supposed that such a narrative, so obviously artificial in character, would have appealed to a very limited audience, and that it would hardly have attracted the curiosity of the "general reader." Yet of the first two editions, issued in 1827 and 1828, 2,500 copies were sold ; there was a third edition in 1884 and a fourth in 1915, in America, and now we have Mr. Peachey's version, handsomely printed, carefully annotated, and embellished by portraits and other illustrations. The truth is that the old type of physician was an interesting and somewhat mysterious figure about whom the world still likes to read. Courtly and dignified of manner, careful and deliberate of speech, he inspired respect and sometimes a rather awful affection. In London, his stately coach, in the country his sturdy roadster, were known of all men, and, in his sedate way, he was as adept at the nice handling of a responsible-looking cane as the young buck was in the flirting of his frivolous, clouded variety. Dr. Radcliffe's cane, crutch-handled in gold, is perhaps the most historic of walking-sticks—if, indeed, it was ever used as a prop ; it was more probably a symbol, a professional ensign. It is of malacca and unusually short When Radcliffe died in 1714 it passed successively to Mead, Askew, Pitcairn, and Baillie, and was presented by the widow of the last-named to the College of Physicians in 1823 ; thus it had a century or more of active work. Its owners were all distinguished men who attended the famous and brought into the world others destined to be equally famous, and the story of its ownership is preserved in the arms of each of its possessors engraved upon it. The book is still eminently readable, full of entertaining and curious matter the interest of which is by no means confined to those concerned with medicine. Mr. Peachey has added a number of elucidatory notes which add greatly to the attraction of the narrative, and high praise must be given to the excellence of the full-page photogravure portraits. It is a handsome book which would adorn the shelves of any medical man who takes a pride in the story of his great predecessors in the first of professions.

### AT THE PARTING OF THE WAYS.

**Persian Sketches.** By the Right Rev. T. H. LINTON, D.D., Bishop in Persia. (Church Missionary Society; 2s. 6d.)

"Persia is at the parting of the ways." This is the opinion of Brigadier-General Sir Percy Sykes, who contributes an Introduction to this very readable little book. "Either matters will go from bad to worse, or there will be a change for the better ; but in any case the time is short. Under these circumstances a book of 'sketches' written by one who has been in close touch with all classes for many years is opportune. Bishop Linton has grasped the Persian character, with its lights and shades, with its good points and its bad. He has also travelled far and wide, and has had the great advantage of securing accurate information on the women through a lady who is probably a medical missionary. Moreover, he possesses the true traveller's spirit, and makes light of hardships and dangers that were very real. The result is a work that merits study, not only by those interested in missions, but also by the statesman, the official and the general public." Sir Percy Sykes thinks that the only thing that can save Persia is "an entire change of outlook among the grandees." The social conditions are indeed appalling, though, as the Bishop points out, "before we pass judgment let us call to mind English social conditions." Venereal disease is one of the scourges of Persia :—

"Here [in the Persian mountains] are two powerful warriors laid low with gonorrhœal joints. . . . Here is a splendid little fellow of eleven with his vocal chords almost destroyed by syphilis. It was through no fault of his own that he was attacked by this fell disease. 'Has anyone in the house got it ?' we asked, and they produced a boy of five with his palate perforated. And so the sad tale goes on, all the more sad because so much of it is caused through no fault of the victim. It is called the 'common or well-known disease,' owing to its prevalence. 'Have you got the common disease ? I asked a patient. 'Of course I have,' was the immediate response. It is ghastly."

The smoking of opium is "a serious danger to the virility of Persia," and tuberculosis is making extraordinarily rapid strides, especially among the nomadic peoples. The conditions of child labour, too, are often very terrible. A doctor of the C.M.S., who has worked for the amelioration of these conditions, writes :—

"Poor parents take their children to the master weavers and accept a contract for the children to serve on receiving a pittance in cash. The children are then compelled to weave, unless absolutely incapacitated by broken-down health. Starting at the age of five, six or seven years, the children, boys and girls alike, work from sunrise to sunset, week in, week out, with but half a day on Friday for a holiday. The work is carried on in ill-ventilated hovels, warmed in the cold of winter only by the heat of their bodies, and overheated in summer through sheer want of space. Thus many fall victims to a very crippling form of late rickets, which affects not only the bones of the arms and legs, but those of the skeleton of the body also. One result is a gross form of knock-knee, which renders walking difficult or impossible, so that many children have to be carried by their parents to and from their looms. There is another result. Girls after their marriage are thrown into the greatest danger as the time of their confinement draws near, both mother and child being faced with certain death."

In spite of these horrors, Dr. Linton writes hopefully of the future. The Persians are anxious to improve conditions, "but improvement to be permanent must be carried out along two lines: First, immediate help for the sufferers such as can be given in a hospital, and by trained welfare workers; and, second, some sort of campaign to arouse and educate Persian public opinion on the whole question of child welfare."

### FASHIONS IN CRIMINOLOGY.

**The Psychology of the Criminal.** By M. Hamblin Smith, M.D. Pp. viii.; 182. (Methuen; 6/- net.)

There are fashions in criminology as in all things else. At one time it is the fashion to blame heredity and at another environment. Then, as to methods of investigation, we may favour physiology or, on the other hand, psychology. Of late psycho-analysis has provided yet another key to unlock the secrets of the criminal mind. It may seem strange that when there have been so many apparently adequate explanations it should still be necessary to look for others. Probably much the same will be said when a few more years, and many more hypotheses, have receded into the past. Meantime Dr. Hamblin Smith has done his best to point the way to the lair of the most deeply buried "complex." There it lies like some maleficent octopus, with its tentacles reaching out towards the surface, charged with quantities of poisonous materials or emotions. For he is a whole-hearted follower of Freud, even to the point of declaring that "there seems to be no doubt as to the supreme importance of the sex instinct." On the other hand, it must be made clear that he does not fail to see that "the theories of the 'newer' psychology are after all only hypotheses; and they are accepted by many of those who have studied them, because they appear to fit the facts better than do other hypotheses."

The danger of so doctrinaire an outlook leading to very unpractical conclusions is obvious. Dr. Hamblin Smith is, however, too closely in touch during his daily work with actuality for this danger to be serious. He insists upon the necessity of considering the individual criminal instead of lumping many offenders into categories. What we need, he thinks, is the introduction of scientific humanitarianism; it is "not punishment, but treatment" that we have to think about when we consider the criminal. It does not greatly matter which path we take so long as we arrive at that desirable end. It is not a matter of sentimentalising over misfits—though that is possibly preferable to brutality. We should endeavour to make "offenders into good citizens on release"—an often difficult, but as experience shows, by no means impossible achievement. But to bring it about we require, even more than the elaborate and comprehensive formulæ for investigating the mind of the criminal set forth in this volume, the sympathy with erring humanity which so evidently inspires its author.

### MISCELLANEOUS NOTICES.

### The Order of St. John.

A Short History of the Order of St. John of Jerusalem. By E. M. Tenison. (Society of SS. Peter and Paul, 32 George Street, Hanover Square, W.1. 3s. 6d. net.) This is a revised and enlarged edition of the short history then called "Chivalry and the Wounded," which was published in October, 1914. The new matter carries the story down to 1918 and to the growth of the Priory for Wales. It is a lively and well-condensed narrative, printed with the care that distinguishes the publications of this society. We hope that the welcome which the book first received may be extended to this new edition.

### A Medical Novelist.

The Braganza Necklace.—By Herbert Harrison. (Sampson Low. 6s. net.)—It is not often that a doctor writes a novel, but in this case Dr. Herbert Harrison shows that he can combine historical knowledge with skill in telling a story. The novel deals with the days of George II. The atmosphere of the reign is very happily suggested, and the story moves with a swing which prevents the interest from ever flagging.

### Dispensing Without Tears.

Dispensing Made Easy. By W. G. Sutherland, M.B., revised by A. L. Taylor, M.P.S. John Wright & Sons, Bristol. Fifth edition. 6s. net.—A useful handbook compiled by an expert, and full of helpful hints upon the art of dispensing with ease and economy. The little book should prove a very sound investment, since by its use the practitioner can save time, trouble and money without loss of efficiency.

### The "Lying-In Hospital."

A Short History of the City of London Maternity Hospital. By Ralph B. Cannings, Secretary.—This interesting pamphlet of forty pages sketches the history of the Lying-in Hospital as it was called till 1918, from its foundation in 1750 to the present day. The old minute books, some of which are unfortunately lacking, provide most of the materials and include many quaint points. It remained only one year in London House, Aldersgate Street, when it moved to Shaftesbury House in the same thoroughfare, which was its home till 1770. It then transferred to City Road; the present institution was built in 1907. The first matron was appointed at £15 a year, a sum equal to about eight times the amount in modern money. An early porter supplemented his earnings by keeping hogs in the backyard, and after their removal was ordered, cultivated guinea-pigs. These, too, were banished, though the Matron was allowed "to keep some pigeons during the pleasure of the committee." She presented the hospital in 1790 with a silver chalice and paten that are still in use. A strange custom which endured till 1791 was that by which the patients were expected to provide tea and sugar for the nurses. Till 1830 the chimneys were swept by climbing boys. In 1888 single women with their first child were admitted.

The tube railway running beneath the City Road finally led to the decision to build a new hospital. During the war the local residents sought refuge in the hospital from air raids. The institution is cramped at present, and Mr. Cannings' history ends with the proposals for fresh additions to the present building. His booklet, which contains several illustrations, is full of interest and agreeably written. It is a welcome addition to the still too few hospital histories that we possess.

### Birth Control.

CONCEPTION CONTROL. By Florence E. Barrett, C.B.E., M.D. Murray. 2s. net.—Lady Barrett holds that this subject should be considered and discussed by the medical profession, and not left to the exploitation of laymen and laywomen. She writes with sincerity and candour upon a subject that is notoriously difficult. She discountenances the use of contraceptive measures other than restriction of intercourse to the pre-menstrual period during which conception is unlikely to occur. The book makes a strong plea for more children in the families of the intellectual classes on economic grounds, and its views are endorsed in a preface by the Archbishop of Canterbury.

### Electric Treatment.

ELECTRIC IONISATION: A Practical Introduction to its use in Medicine and Surgery. By A. R. Friel, M.A., M.D., F.R.C.S.I. Wright, Bristol.—This useful little book makes a welcome appearance in its second edition. The author has put before us very exactly what can be expected from ionisation and what cannot. This in itself is a recommendation to the busy practitioner, but for those more favoured with time and opportunity for experiment, Dr. Friel has provided sufficient technical detail to make an excellent little manual. In the new edition have been introduced special chapters on pyorrhœa, endometritis and otorrhœa as dealt with by ionisation. Ample attention is now given to the essential electrical facts and the explanation of electrical terms, and many useful clinical hints are included.

### A Manual for the Layman.

RHEUMATISM AND GOUT. By Dorothy C. Hare, C.B.E., M.D., M.R.C.P. (The Scientific Press. 1s. 3d. net.)—This is a useful little book explaining in as simple terms as possible the exceedingly complicated diseases of rheumatism and gout. In addition to an introduction on bacterial diseases and toxins, there are some anatomical notes, chapters on acute rheumatism, arthritis and muscular rheumatism, and one chapter on gout. This booklet should be of peculiar moment to the layman, for, as Dr. Hare points out, " the subject of rheumatism is not only of interest to the general reader, or of professional interest to the nurse or health worker, it is a matter of practical importance that the public should be better instructed."

### The Newest Lights on Diabetes.

MODERN METHODS IN THE DIAGNOSIS AND TREATMENT OF GLYCOSURIA AND DIABETES. By Hugh Maclean. Constable. 12s.—This monograph can be recommended heartily to those who wish to keep abreast of recent knowledge of diabetes. The book is clearly written and well printed; it treats the subject from the bio-chemical standpoint, but the clinical bearings of chemical investigations are indicated, and there are short but lucid instructions for treatment. The value of blood examination for sugar and alkalinity is emphasised, and the methods of carrying out these investigations are clearly described. The author points out that intravenous injection of bicarbonate in cases of coma is useless, and may be harmful. This is a useful book, and further editions will certainly be demanded as knowledge increases; meanwhile it is completely up-to-date.

### A Nursing Annual.

THE NURSING MIRROR POCKET ENCYCLOPÆDIA AND DIARY. The Scientific Press. 1s. 6d. net. The 1923 issue of this excellent little book of reference has been carefully revised, and contains much new matter and many additional illustrations. It is now in its sixteenth year of issue, and grows steadily in usefulness and popularity.

### Junior Chemistry.

CHEMISTRY FOR BEGINNERS AND SCHOOLS. By C. T. Kingzett, F.I.C., F.C.S. Fourth Edition, with Glossary. (Baillière, Tindall & Cox. 5s. net.) The author of the well-known " Dictionary of Chemistry " has prepared a fourth edition of his popular manual, which will be welcomed by teachers and students alike. In the Glossary, which has been considerably enlarged, is a complete list of all the elements, with their symbols and international atomic weights (1921). This list contains the names of the rare earths, lutecium, yttrium, praseodymium, &c. That the author believes in developing a broad outlook in his readers is evident by his inclusion of " Septic Poisons " in his Glossary. As an introductory text-book of chemistry this book will continue to hold a foremost position.

### Anatomy of the Bronchial Glands.

L'ADENOPATHIE TRACHES-BRONCHIQUE SIMPLE CHEZ L'ENFANT. By Docteur Henri Forgeson. (Librairie Littéraire et Médicale, Louis Arnette, Paris.) This monograph is a thesis presented at the University of Paris. It consists of an account of the anatomy of the bronchial glands, and descriptions of the lesions found in them in a number of necropsies conducted by the author. It is designed to prove that inflammatory and other pathological changes occur in the bronchial glands of children without the activity of the tubercle bacillus. The writer suggests that in some cases of combined pulmonary and glandular disease the latter plays the primary part. While the thesis is mildly interesting, it contains nothing new, and the reader is unlikely to share the enthusiasm of the author for his subject.

### Ear, Nose and Larynx.

OTO-RHINO-LARYNGOLOGY. By George Laurens. Authorised English translation of the fourth revised French edition, by H. Clayton Fox, F.R.C.S.I. Preface by Sir J. Dundas-Grant, M.A., M.D., F.R.C.S. John Wright & Sons, Bristol.—This is the second English edition of a book which by its somewhat unaccustomed title might at once be recognised as of Continental origin. This fact need, however, in no way deter an English reader. The translation is extremely well done, and the matter is of a strikingly practical nature. This particular subject is one in which detail, clinical and technical, is peculiarly abundant, and tends therefore, in a large number of otherwise admirable works, to obscure the main issues. The best work for the general practitioner's use is obviously one in which every small fact is noted, but strictly according in its average and actual importance. The author's stated object is not to deal with every disease of the ear, nose and larynx, but rather to show how the conditions which are most commonly seen in practice may be recognised. This Dr. Laurens achieves, and in doing so has given us a book of considerable utility.

### The Psycho-Analyst in the Pillory.

" SUGGESTION " AND COMMON SENSE. By R. Allan Bennett, M.D., M.R.C.P. Pp. 105. (John Wright & Sons. 6s. net.)—We do not feel that we learn much that is new from this book, either as regards methods or results. The author attacks the psycho-analytic school, and accuses its members of treating " a fragile and elusive figment with a gross and unsatisfying materialism." Freud, or Jung, or any other exponent of the newer psychological methods, would be the first to admit that his conception of the unconscious is but a hypothesis, adopted because it appears to account better than any other for certain phenomena which have been studied on scientific lines. Dr. Bennett, in spite of his expressed dislike for psychology, enters into metaphysics. He endows every unicellular organism, and each individual cell in the human body, with intelligence. We have no quarrel with this view. It would appear to follow from the teaching of W. K. Clifford, and may, indeed, be traced back to Leibnitz. The remarks on this head are of interest, and are worthy of consideration in view of the light which such a hypothesis throws on certain current biological problems. The subject is, however, treated too briefly to assist us much in our study of suggestion. There is no index, a grave fault even in so small a book.

